# Estimating balance, cognitive function, and falls risk using wearable sensors and the sit-to-stand test

**DOI:** 10.1017/wtc.2022.6

**Published:** 2022-06-07

**Authors:** Barry R. Greene, Emer P. Doheny, Killian McManus, Brian Caulfield

**Affiliations:** 1 Kinesis Health Technologies Ltd, Dublin, Ireland; 2 Insight Centre for Data Analytics, University College Dublin, Dublin, Ireland; 3 School of Electrical and Electronic Engineering, University College Dublin, Dublin, Ireland; 4 School of Public Health, Physiotherapy and Sports Science, University College Dublin, Dublin, Ireland

**Keywords:** balance, Berg balance scale, cognitive decline, falls, inertial sensor, sit-to-stand test

## Abstract

The five times sit-to-stand test (FTSS) is an established functional test, used clinically as a measure of lower-limb strength, endurance and falls risk. We report a novel method to estimate and classify cognitive function, balance impairment and falls risk using the FTSS and body-worn inertial sensors. 168 community dwelling older adults received a Comprehensive Geriatric Assessment which included the Mini-Mental State Examination (MMSE) and the Berg Balance Scale (BBS). Each participant performed an FTSS, with inertial sensors on the thigh and torso, either at home or in the clinical environment. Adaptive peak detection was used to identify phases of each FTSS from torso or thigh-mounted inertial sensors. Features were then extracted from each sensor to quantify the timing, postural sway and variability of each FTSS. The relationship between each feature and MMSE and BBS was examined using Spearman’s correlation. Intraclass correlation coefficients were used to examine the intra-session reliability of each feature. A Poisson regression model with an elastic net model selection procedure was used to estimate MMSE and BBS scores, while logistic regression and sequential forward feature selection was used to classify participants according to falls risk, cognitive decline and balance impairment. BBS and MMSE were estimated using cross-validation with low root mean squared errors of 2.91 and 1.50, respectively, while the cross-validated classification accuracies for balance impairment, cognitive decline, and falls risk were 81.96, 72.71, and 68.74%, respectively. The novel methods reported provide surrogate measures which may have utility in remote assessment of physical and cognitive function.

## Introduction

The chair stand and sit-to-stand (STS) tests are well-established functional tests, used clinically as a measure of lower-limb strength, endurance, and falls risk (Csuka and McCarty, 1985; Whitney et al., 2005). The five times sit-to-stand (FTSS) is a variant of chair stand and STS tests, which is widely used in rehabilitation settings (Whitney et al., 2005). The FTSS has been associated with both balance impairment (Whitney et al., 2005) and cognitive decline (Annweiler et al., 2011). The FTSS has also been shown to correlate with clinical measures of balance and mobility impairment, such as the Berg Balance Scale (BBS) (Berg, 1989) and the Performance Oriented Mobility Assessment scale (Tinetti, 1986; Knobe et al., 2016), which are usually conducted in a clinical environment. However, the FTSS is thought to be suitable for remote, home-based assessment, particularly if test performance can be precisely measured over time (Adamowicz et al., 2020; Park et al., 2021); to that end, the FTSS has been augmented using smartphone and body-worn inertial sensors in previous studies (Zijlstra et al., 2010; Millor et al., 2014; Cerrito et al., 2015; van Lummel et al., 2016; Ejupi et al., 2017; Pham et al., 2018), to enable objective movement analysis. Inertial sensors mounted on the thighs and torso have been used to quantify movement during the FTSS (Janssen et al., 2008; Millor et al., 2014), and to examine associations with falls risk (Najafi et al., 2002; Doheny et al., 2013) and frailty in older adults (Ganea et al., 2011; Galán-Mercant and Cuesta-Vargas, 2013). In addition to wearable or smartphone-based inertial sensors, depth cameras have also been used to quantify the FTSS in the home to remotely assess falls risk (Ejupi et al., 2016). Knobe et al. (2016) examined the mutual correlations of a number of functional assessment tests and found the FTSS could identify balance dysfunction when combined with a selection of other functional tests. Park et al. (2021) examined the use of the FTSS with a wearable sensor to remotely quantify frailty. Similarly, Ganea et al. (2011) showed that an STS test instrumented with thigh and torso inertial sensors was correlated with frailty and Greene et al. (2014) showed that a sit-to-stand test instrumented with inertial sensors could be combined with other functional tests to assess frailty and falls risk.

The association between balance and motor function with cognitive decline in older adults has been well established; however, the nature and causality of the relationship between physical task performance and cognitive function is not as well understood. Changes in motor function and associated decline in physical performance are thought to precede the onset of cognitive impairment (Camicioli et al., 1998; Atkinson et al., 2005; Wang et al., 2006), while gait disturbances and postural instability are reported to be associated with cognitive decline (Rosano et al., 2005). Clinical gait abnormalities have been shown to predict non-Alzheimer’s dementia while gait parameters have been found to predict cognitive decline in non-demented older adults (Verghese et al., 2007). There has been limited reported research on predicting cognitive status using instrumented functional tests. Lo et al. (2019) used a smartphone-based assessment battery which included voice, balance, gait, and reaction time measures, to predict cognitive decline and functional impairment in people with Parkinson’s Disease at 18 months. Suzuki et al. (2020) used waveform similarity analysis on selected segments to estimate Mini-Mental State Examination (MMSE) scores from outdoor walking using a combined GPS and inertial sensor. Previous research by our group (Greene and Kenny, 2012) used wearable sensors and the Timed Up and Go (TUG) test to predict cognitive decline after two years, in a sample of 189 older adults and also reported strong associations between cognitive decline and quantitative gait measures.

Despite this body of work, a system using a single instrumented functional test to provide surrogate measures of cognitive function, balance impairment, and falls risk for use in screening and monitoring has not yet been reported and could be of significant benefit. In addition, the correlation of individual thigh and torso inertial sensor features obtained during the FTSS test with clinical measures of balance and cognitive function, may provide new insights into the interplay between specific movement patterns and clinical measures of cognitive decline and balance impairment.

This study aimed to develop a system to assess balance, cognitive function, and falls risk in older adults using a single functional test. To achieve this, a novel method to estimate clinical measures of balance and cognitive function (the BBS score (Berg, 1989) and the MMSE (Kukull et al., 1994) was developed using torso or thigh inertial measurement unit (IMU) data obtained during the FTSS test. In addition, we investigated the utility of this method in classifying balance impairment, cognitive decline, and falls risk. We also report the intra-session reliability of each thigh and torso inertial sensor feature, as well as the correlation of each calculated feature with the MMSE and BBS scores.

The methods reported here could form the basis of a remote assessment platform, using body-worn or smartphone-based inertial sensors obtained during a functional test (such as the FTSS) to provide surrogate measures of balance, cognitive function, and falls risk.

## Data

One-hundred and sixty-eight community-dwelling older adults completed an FTSS test instrumented with inertial sensors placed on the thigh and torso. The dataset included two sub-studies; sub-study one consisted of 40 participants who completed an FTSS in their homes at four different time points over the course of a single day, while supervised by a researcher. Sub-study two consisted of 128 participants who completed three iterations of the FTSS test in a clinical environment, with at least 1 min rest between each iteration. The data for both of these supervised sub-studies were pooled in order to obtain a larger, more varied, and representative data set.

In conducting the FTSS test, each participant was asked to fully stand up and sit back down five times as quickly as possible. The researcher said ‘go’ when they started the recording, and ended the recording when the participant was re-seated for the fifth time with their back touching the back of the chair. Ethical approval for both studies was obtained from the St James Hospital ethics committee, Dublin, Ireland. Inclusion criteria were: aged 60 and over, with no history of stroke and the ability to provide informed consent.

Each participant also received a Comprehensive Geriatric Assessment (CGA) including visual acuity (Binocular LogMar) and visual contrast sensitivity (Pelli-Robson) tests as well as the Berg Balance Scale (BBS) (Berg, 1989) and the Mini Mental State Examination (MMSE) (Cullen et al., 2005; Kukull et al., 1994.

For the purposes of this analysis, data from both studies were pooled; a total of 75 participants reported a history of falls in the past 5 years, with falls history data recorded in line with AGS/BGS guidelines (Gillespie et al., 2001). Ninety-five participants reported polypharmacy and 44 participants reported orthostatic hypotension. A fall was defined as an event resulting in a person coming to rest on the lower level regardless of whether an injury was sustained, and not as a result of a major intrinsic event or overwhelming hazard (Tinetti et al., 1988). Participants who had experienced more than one fall during the 12 months prior to assessment or had a fall resulting in any injury were categorized as fallers. Participants not meeting these criteria were categorized as non-fallers. Clinical details for both data sets are summarized in Table 1.

**Table 1.** Clinical information for both data sets (supervised in clinic and home environments)

Variable	Supervised home (*N* = 40)	Supervised clinic (*N* = 128)	Combined data set (*N* = 168)
Gender (M/F)	17/23	34/94	51/117
Height (cm)*	168.5 ± 9.5	161.5 ± 8.2	163.1 ± 9.0
Weight (kg)*	76.9 ± 11.3	72.1 ± 13.0	73.2 ± 12.7
Age (yrs)*	71.4 ± 7.3	76.1 ± 6.8	75.0 ± 7.2
Polypharmacy (*N*)*	17	78	95
MMSE	28.4 ± 1.6	27.9 ± 2.1	28.0 ± 2.0
Grip (lbs)*	61.0 ± 23.7	48.0 ± 19.6	51.1 ± 21.3
Impaired visual fields (*N*)*	1	20	21
Orthostatic hypotension (*N*)*	19	25	44
BBS	53.0 ± 4.4	52.8 ± 4.4	52.9 ± 4.4
Walking aid (*N*)*	4	100	104
Falls history (Faller/Non-faller) (*N*)	20/20	61/67	75/93

*Note.* Significant differences (*p* < .05, from Chi-square or Wilcoxon rank-sum tests) between supervised home and supervised clinic data sets are indicated by an asterisk.

Abbreviations: BBS, Berg balance scale; MMSE, Mini-Mental State Examination.

**Figure 1.**
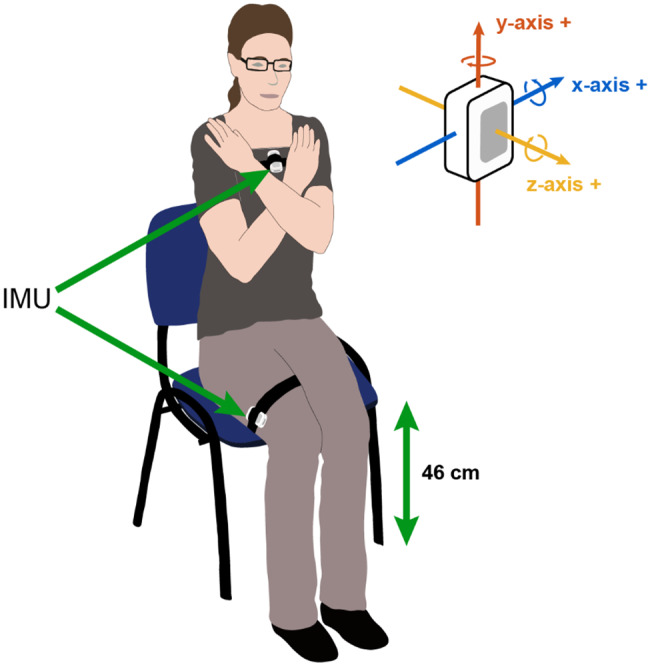
Experimental setup for FTSS test. Inertial sensors are secured to the thigh and torso.

Each FTSS test was instrumented by IMUs placed on the thigh and torso (Shimmer2R, Shimmer Research, Dublin, Ireland), see Figure 1. The thigh sensor was secured along the lateral aspect of the thigh using an elasticized bandage, while the torso sensor was secured to the sternum using a Velcro strap. Each IMU consisted of a tri-axial accelerometer and a tri-axial gyroscope. IMU data were synchronized and aggregated using a dedicated software application (Kinesis Health Technologies, Dublin Ireland) running on an Android tablet. Raw inertial sensor data were sampled at 102.4 Hz, calibrated using a standard method (Ferraris et al., 1995), and bandpass filtered in the range 0.01–5 Hz (Ferraris et al., 1995), using a Butterworth IIR digital filter.

## Method

### Sensor Signal Processing

Two signal processing algorithms were developed to quantify the FTSS test using inertial sensors mounted on either the torso or the thigh. Each algorithm identifies phases of each sit-to-stand transition within the test as well as quantifying sway and movement quality, using a range of features listed in Table 2, during the FTSS.

**Table 2.** Reliability and correlation analysis for pooled data set of torso and thigh features across all FTSS tests

		Torso sensor				Thigh sensor			
Variable name	Technical name	ICC (95% CI)		MMSEρ	BBSρ	ICC (95% CI)		MMSEρ	BBSρ
TotalTime	Time to complete FTSS	0.86	(0.82−0.90)	−0.11	−0.26	0.86	(0.81−0.89)	−0.09	−0.39
MeanSSSTime	Mean sit-stand-sit time	0.74	(0.67−0.80)	−0.15	−0.47	0.50	(0.35−0.62)	−0.08	−0.42
CVSSSTime	Coefficient of variation of sit-stand-sit time	0.30	(0.10−0.47)	−0.06	−0.17	0.57	(0.45−0.67)	0.12	−0.01
NumReps	Number of sit-to-stand transitions completed	0.84	(0.80−0.88)	0.00	0.15	0.83	(0.79−0.87)	0.09	0.07
MeanStandTime	Mean time to stand-up	0.83	(0.78−0.87)	−0.11	−0.40	0.36	(0.17−0.51)	0.07	−0.17
CV_StandTime	Coefficient of variation of stand time	0.50	(0.35−0.61)	−0.09	−0.22	0	(0−0.21)	−0.01	−0.09
MeanSitTime	Mean time to sit down	0.58	(0.46−0.68)	−0.14	−0.43	0.39	(0.21−0.53)	0.08	0.01
CV_SitTime	Coefficient of variation of sit time	0.30	(0.10−0.47)	−0.03	−0.16	0.33	(0.14−0.49)	0.10	−0.01
Fmed_Acc	Median frequency acceleration	0.71	(0.62−0.78)	0.08	0.25	0.54	(0.41−0.65)	0.09	0.15
SEF_Acc	Spectral edge frequency acceleration	0.66	(0.56−0.74)	0.10	0.15	0.74	(0.66−0.80)	0.07	0.15
H_Acc	Spectral entropy acceleration	0.70	(0.62−0.77)	−0.01	−0.19	0.80	(0.74−0.85)	0.11	0.14
Fmed_AngVel	Median frequency angular velocity	0.65	(0.55−0.74)	0.24	0.19	0.78	(0.72−0.84)	0.13	0.21
SEF_AngVel	Spectral edge frequency angular velocity	0.73	(0.65−0.80)	0.12	0.14	0.78	(0.71−0.83)	0.06	0.12
H_AngVel	Spectral entropy angular velocity	0.67	(0.57−0.75)	0.10	−0.01	0.81	(0.75−0.85)	0.09	0.01
AccZ_stand_start_mean	Mean *Z*-axis acceleration at stand-start	0.87	(0.83−0.90)	0.21	0.16	0.91	(0.88−0.93)	−0.14	−0.35
AccZ_sit_end_mean	Mean *Z*-axis acceleration at sit-end	0.85	(0.81−0.89)	0.08	0.15	0.91	(0.89−0.93)	−0.14	−0.35
AccZ_mid_stand_mean	Mean *Z*-axis acceleration at mid-stand	0.65	(0.54−0.73)	−0.07	−0.04	0.95	(0.94−0.96)	−0.12	−0.27
AccZ_stand_start_std	Std. dev. of *Z*-axis acceleration at stand-start	0.60	(0.48−0.69)	0.07	0.03	0.78	(0.71−0.83)	0.08	0.07
AccZ_sit_end_std	Std. dev. of *Z*-axis acceleration at sit-end	0.56	(0.44−0.67)	0.04	0.06	0.73	(0.65−0.79)	−0.05	0.06
AccZ_mid_stand_std	Std. dev. of *Z*-axis acceleration at mid-stand	0.56	(0.44−0.67)	0.07	0.01	0.76	(0.69−0.82)	0.05	0.04
AV_X_stand_start_mean	Mean *X*-axis angular velocity at stand-start	0.89	(0.85−0.91)	−0.11	−0.11				
AV_X_sit_end_mean	Mean *X*-axis angular velocity at sit-end	0.67	(0.58−0.75)	0.12	0.27				
AV_X_mid_stand_mean	Mean *X*-axis angular velocity at mid-stand	0.31	(0.10−0.47)	0.05	0.11				
AV_X_stand_start_std	Std. dev. of *X*-axis angular velocity at stand-start	0.65	(0.54−0.73)	−0.01	0.01				
AV_X_sit_end_std	Std. dev. of *X*-axis angular velocity at sit-end	0.63	(0.53−0.72)	0.07	−0.03				
AV_X_mid_stand_std	Std. dev. of *X*-axis angular velocity at mid-stand	0.62	(0.51−0.71)	0.01	0.14				
RMS_X_Total_Acc	Root mean square acceleration (*X*)	0.94	(0.92−0.95)	−0.12	0.08				
RMS_Y_Total_Acc	Root mean square acceleration (*Y*)	0.90	(0.88−0.93)	0.12	0.09				
RMS_Z_Total_Acc	Root mean square acceleration (*Z*)	0.93	(0.91−0.95)	0.18	0.18				
Jerk_X_Total_Acc	Jerk acceleration (*X*)	0.50	(0.36−0.62)	0.04	−0.13				
Jerk_Y_Total_Acc	Jerk acceleration (*Y*)	0.42	(0.25−0.56)	−0.05	−0.01				
Jerk_Z_Total_Acc	Jerk acceleration (*Z*)	0.47	(0.32−0.60)	0.07	0.08				
RMS_X_Total_AV	Root mean square angular velocity (*X*)	0.94	(0.92−0.95)	0.03	0.00				
RMS_Y_Total_AV	Root mean square angular velocity (*Y*)	0.91	(0.89−0.93)	0.06	0.05				
RMS_Z_Total_AV	Root mean square angular velocity (*Z*)	0.96	(0.95−0.97)	0.06	0.20				
Jerk_X_Total_AV	Jerk angular velocity (*X*)	0.15	(0.09−0.35)	0.03	0.04				
Jerk_Y_Total_AV	Jerk angular velocity (*Y*)	0.31	(0.11−0.47)	0.09	0.07				
Jerk_Z_Total_AV	Jerk angular velocity (*Z*)	0.44	(0.27−0.57)	0.15	0.32				

*Note.* ICC(2,k) with 95% confidence intervals and Spearman’s rank correlations against MMSE (MMSEρ) and BBS (BBSρ) are reported.

Abbreviations: BBS, Berg balance scale; MMSE, Mini-Mental State Examination.

**Figure 2.**
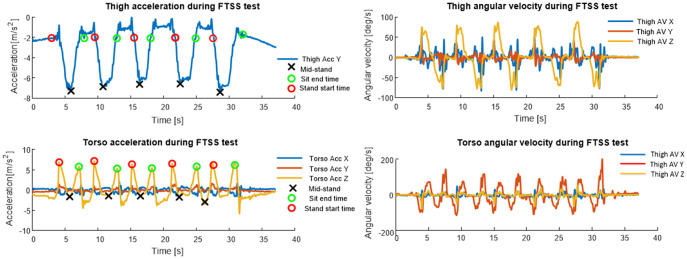
Thigh and torso IMU signals for an FTSS test. Automatically detected fiducial points (mid-stand, sit-end time, and stand start time) on thigh and torso accelerometer signals are highlighted.

Using the accelerometer data recorded by the torso sensor, adaptive peak detection was applied to the sensor *Z*-axis acceleration (*⍺*_z_) to identify the times associated with stand start, mid-stand and sit-end. Positive and negative peaks in the signal were detected using the thresholds 0.4*⍺*_z-max_ and 0.5*⍺*_z-min_, where *⍺*_z-max_ and *⍺*_z-min_ are the minimum and maximum absolute valued *Z*-axis acceleration points, respectively. A step-back search was employed to ensure peaks are cleanly detected. Maximum acceleration peaks were used to identify stand start or sit-end points, with negative points between a pair of stand start and sit-end points corresponding to mid-stand points (see Figure 2). These peaks were chosen as the morphological signal elements most suitable for reliable automatic detection of sit-to-stand and stand-to-sit transitions.

The accelerometer data recorded by the thigh sensor was processed using a modified version of the method reported by Doheny et al. (2013), which uses peak detection applied to femoral (*Y*-axis) acceleration to identify mid-stand, sit-end, and stand start times. The minimum *Y*-axis acceleration over the total FTSS, *⍺*_y-min_, was used to detect the minimum acceleration during each Sit Stand Sit (SSS) phase, referred to as mid-stand points. Only mid-stand points with accelerations, *⍺*_ms_, less than 0.8*⍺*_y-min_ were deemed successful SSS attempts, to ensure that each successful SSS phases were detected. For both thigh and torso sensor algorithms, signal validity checks were incorporated to remove any movement artifact (large spikes) from the signal, to confirm the correct sensor position (skewness of signal) during the test, and to examine the signal for dropped or missing samples, while the first second of data are removed from each trial to exclude any spurious data due to sensor-settling.

Features quantifying the timing and sway characteristics of individual sit-to-stand transitions were calculated from the calibrated accelerometer and angular velocity signals. The root mean square (RMS) and jerk (a derivative of the acceleration and angular velocity signals) were calculated from the acceleration and angular velocity signals of the torso sensor to quantify sway during the FTSS, however, they were not calculated for the thigh sensor as it was felt a thigh-mounted sensor could not reasonably measure upper body postural sway.

### Exploratory Analysis

Spearman’s rank correlation was used to examine the relationship between each feature with MMSE and BBS while Wilcoxon’s rank-sum test was used to test for differences between fallers and non-fallers for each feature, with data for each participant were averaged across all trials. Wilcoxon rank-sum was also used to test for significant differences in continuous clinical and demographic measures between the supervised home and supervised clinical datasets, while a Chi-square test of proportions was used to examine differences between the two data sets for categorical variables (see Table 1).

Intraclass correlation coefficients (ICC(2,k)) were used to examine the intra-session reliability of all features for both sensor locations. Reliability values above 0.90 were considered ‘excellent’, values greater than 0.75 were considered ‘good’, while reliability in the range of 0.5 and 0.75 was ‘moderate’. ICC values less than 0.5 was considered poor reliability (Koo and Li, 2016).

### Classification Analysis

To determine how well IMU features calculated during the FTSS test could assess balance, cognitive function and falls risk, a logistic regression classifier model with sequential forward feature selection was used and validated using 10-fold cross-validation. For each classifier model only the first assessment per participant was included in the analysis, so that algorithm results are comparable to a scenario based on a single functional test assessment.

The MMSE data were dichotomized into cognitively impaired and cognitively intact with values below a threshold of 27 used to identify impaired cognition (Kukull et al., 1994. Similarly, BBS data were dichotomized into impaired and normal balance, with a score below a threshold of 53 denoting impaired balance (Muir et al., 2008). Falls risk was classified using falls history, with each participant labeled as a faller or non-faller based on their history of falls in the previous 12 months.

For each classifier model, feature selection was performed within a cross-validation procedure to avoid bias, interaction terms were included in each feature set and each model was constrained to include the time to complete the FTSS test. Signal features were extracted for the first assessment for all 168 participants. Each feature per participant along with their age, gender, height, and weight was made available for model selection. Separate models per sensor location (torso or thigh) were considered, along with separate male and female models per sensor location.

#### Classifier performance metrics

A number of measures were used to evaluate the performance of each classifier model. Taking the MMSE model as an example, the classification accuracy (Acc) is defined as the percentage of participants correctly classified by the system as being cognitively declined or cognitively intact. The sensitivity (Sens) is defined as the percentage of cognitively declined participants classified correctly. Similarly, specificity (Spec) is defined as the percentage of participants, cognitively intact at baseline correctly identified as such by the system. Positive and negative predictive values were also calculated to provide a measure of the predictive power of positive and negative (cognitively impaired or cognitively intact) classifications. The positive predictive value (PPV) is defined as the proportion of participants, classified as cognitively impaired by the algorithm, correctly classified. Similarly, the negative predictive value (NPV) is the proportion of participants, classified as cognitively intact by the algorithm, who are correctly classified. The same metrics were also used to evaluate the balance impairment and falls risk classification performance.

### Regression Analysis

Poisson regression with an elastic net model selection procedure was used to develop models to estimate BBS and MMSE scores. Each model was validated using 10-fold cross-validation. The elastic net alpha value set a priori to 0.1, and models were also a priori constrained to a minimum model size (number of features) of three and a maximum model size of 20. Each model based on FTSS sensor parameters was constrained to include the time to complete the FTSS test (total time). As with the classification analysis, separate models per sensor location were considered along with separate models for male and female participants. Each feature (see Table 1) along with age, sex, height, and weight was made available for the model selection procedure.

#### Regression model performance metrics

The performance of each regression model on the test sets was evaluated using the following metrics: coefficient of determination (*R*^2^), root mean squared error (RMSE), Spearman’s rank correlation (*ρ*), and model size (number of features). All signal processing and statistical analysis were conducted offline using Matlab (Mathworks, Richmond, VA).

## Results

### Exploratory Analysis

The mean MMSE score was 28.0 ± 2.0, while the mean BBS score was 52.9 ± 4.4. Moderate correlations were evident between BBS/MMSE and a number of individual features derived from the thigh and torso sensors, including total time, mean sit-stand-sit time, measures based on jerk, and those based on the acceleration during the stand phase. No significant differences (*p* < .05) were found between fallers and n,on-fallers for features obtained from either torso or thigh sensors.

Reliability analysis of the pooled data set found that 12 of 38 torso features calculated from the torso sensor showed good reliability (ICC > 0.75) with excellent reliability (ICC > 0.9) found for 6 of 38; Poor reliability (ICC < 0.5) was found for 9 of 38 features. For the thigh sensor, 11 of 20 features were found to be reliable while poor reliability was observed for 5 of 20 features (Table 2). For both sensor locations, features based on coefficients of variation and jerk obtained from the torso sensor exhibited lower reliability than those based on average values as previously reported (Doheny et al., 2013).

### Classification Analysis

For analysis of falls risk, 81 participants reported a history of falls in the past 12 months and were labeled as fallers, while 87 reported no falls and were labeled as non-fallers. Fifty-three of 168 participants were found to have impaired balance (based on BBS threshold of 53) while 48 of 168 participants were found to be cognitively impaired (based on MMSE threshold of 27).

Results for classification of balance, cognitive function and falls risk using either thigh or torso mounted sensors during the FTSS as detailed in Table 3 below. Details on the model coefficients and selected features for each of the classifier models are provided in the Supplementary Material.

**Table 3.** Classification results for assessment of balance, cognitive function and falls risk using either thigh or torso mounted sensors during the FTSS

	Torso sensor BBS model				Torso sensor MMSE model				Torso sensor Falls model			
Condition	All	M	F	Mean	All	M	F	Mean	All data	M	F	Mean
Acc (%)	76.76	86.36	77.55	81.96	66.90	72.73	70.41	71.57	66.20	77.27	60.20	68.74
Sens (%)	46.34	25.00	36.36	30.68	29.79	61.11	31.03	46.07	60.29	71.43	59.57	65.50
Spec (%)	89.11	100.00	98.46	99.23	85.26	80.77	86.96	83.86	71.62	82.61	60.78	71.70
PPV (%)	63.33	100.00	92.31	96.15	50.00	68.75	50.00	59.38	66.13	78.95	58.33	68.64
NPV (%)	80.36	85.71	75.29	80.50	71.05	75.00	75.00	75.00	66.25	76.00	62.00	69.00

	Thigh sensor BBS model				Thigh sensor MMSE model				Thigh sensor Falls model			
Condition	All	M	F	Mean	All	M	F	Mean	All	M	F	Mean
Acc (%)	81.69	80.95	75.00	77.98	68.31	71.43	74.00	72.71	54.23	66.67	64.00	65.33
Sens (%)	51.22	50.00	39.39	44.70	18.75	44.44	40.00	42.22	48.57	72.73	62.50	67.61
Spec (%)	94.06	88.24	92.54	90.39	93.62	91.67	88.57	90.12	59.72	60.00	65.38	62.69
PPV (%)	77.78	50.00	72.22	61.11	60.00	80.00	60.00	70.00	53.97	66.67	62.50	64.58
NPV (%)	82.61	88.24	75.61	81.92	69.29	68.75	77.50	73.13	54.43	66.67	65.38	66.03

*Note.* Results are evaluated using Accuracy (Acc), Sensitivity (Sens), and Specificity (Spec) as well as Positive and Negative Predictive value (PPV and NPV, respectively). M and F refer to male and female models, respectively, while, Mean refers to the performance averaged across male and female models.

Abbreviations: BBS, Berg balance scale; FTSS, five times sit-to-stand test; MMSE, Mini-Mental State Examination.

### Regression Analysis

Results for regression models of MMSE and BBS scores using either torso or thigh inertial sensors are reported in Table 4. Given the significant impacts of gender on the outcomes of interest (Fried et al., 2001; Steffen et al., 2002; Chang and Do, 2015), we report separate results for models based on male and female data only as well as for models based on both male and female data.

**Table 4.** Cross-validated elastic net regression results, for models of BBS and MMSE based on thigh or torso mounted IMUs during the FTSS test

Condition	Torso sensor BBS model					Torso sensor MMSE model				
Model	*N*	*R*^2^	RMSE	*ρ*	DoF	*N*	*R*^2^	RMSE	*ρ*	DoF
All	168	0.45	3.17	0.68	20	168	0.31	1.60	0.55	16
Male	51	0.74	2.53	0.71	20	51	0.22	1.71	0.60	9
Female	117	0.57	2.60	0.70	17	117	0.34	1.53	0.54	11
Mean (Male/Female)		0.65	2.57	0.70	20		0.28	1.62	0.57	11

Condition	Thigh sensor BBS model					Thigh sensor MMSE model				
Model	*N*	*R*^2^	RMSE	*ρ*	DoF	*N*	*R*^2^	RMSE	*ρ*	DoF
All	168	0.29	3.76	0.58	5	168	0.11	1.83	0.37	2
Male	51	0.27	4.66	0.66	5	51	0.21	1.72	0.55	6
Female	117	0.51	2.82	0.61	20	117	0.27	1.63	0.50	19
Mean (Male/Female)		0.39	3.74	0.63	20		0.24	1.67	0.52	19

*Note.* Results are evaluated using coefficient of determination (*R^2^*), root mean square error (RMSE), Spearman’s rank correlation (*ρ*), and the number of features in the model (DoF). The number of participants included in each analysis (*N*) is also listed.

Abbreviations: BBS, Berg balance scale; FTSS, five times sit-to-stand test; IMU, inertial measurement unit; MMSE, Mini-Mental State Examination.

Figures 4 and 5 show the calculated value of each score plotted against the actual value for all data, as well as male and female models.

**Figure 3.**
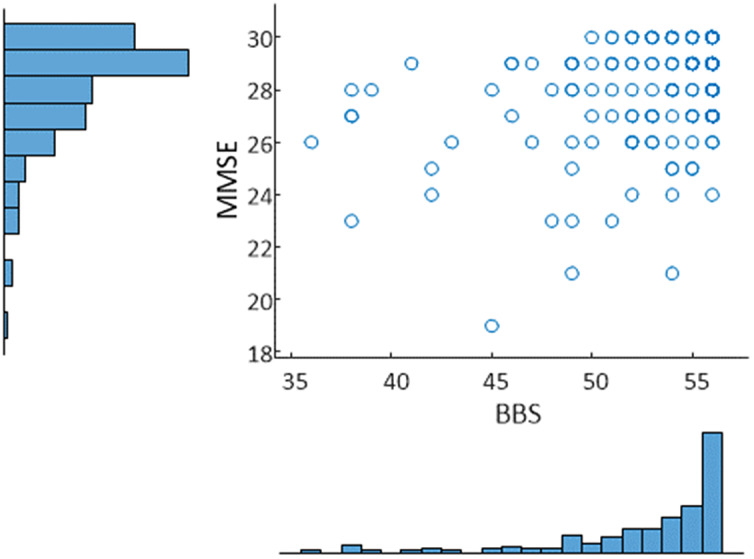
Relationship between BBS and MMSE along with histograms of each distribution for the sample. Correlation: 0.35. A threshold of 53 was used to identify balance impairment from BBS, while a threshold of 27 was used to identify cognitive decline from MMSE.

**Figure 4.**
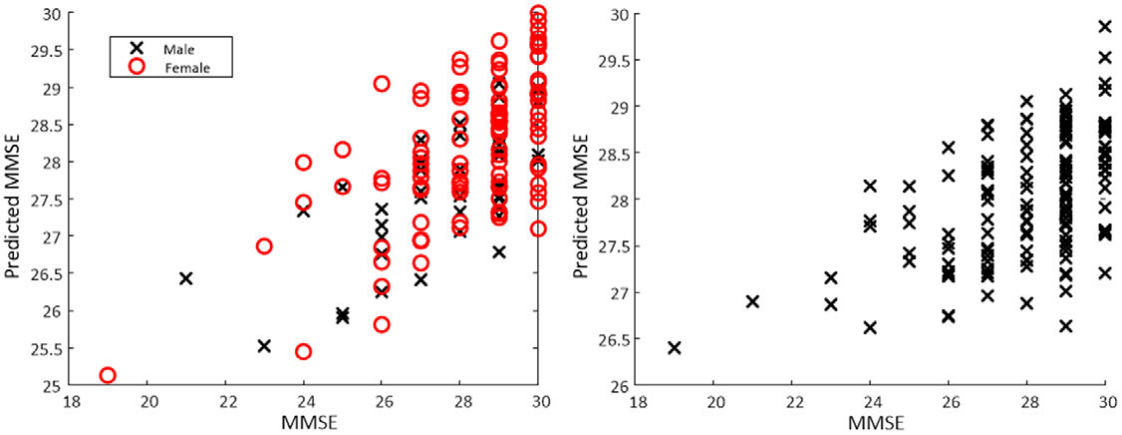
Scatter plot of actual MMSE versus predicted MMSE based on a regression model obtained from a torso-mounted IMU during the FTSS. For the separate male/female models (left panel) mean *R*^2^ was 0.28 with mean RMSE of 1.62. For the all data model (right panel) *R*^2^ of 0.31 and RMSE of 1.60 was observed.

**Figure 5.**
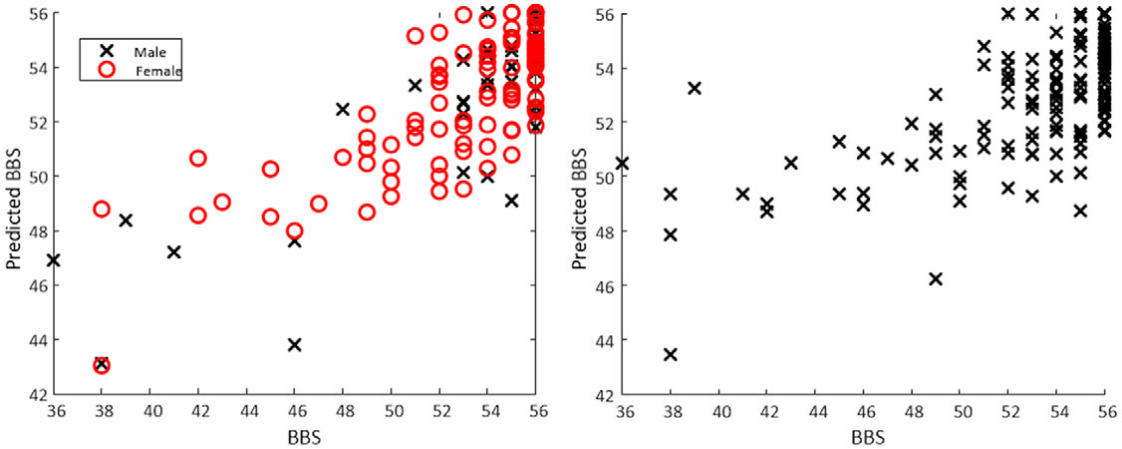
Scatter plot of actual BBS versus predicted BBS based on a regression model obtained from a torso-mounted IMU during the FTSS. For the separate male/female models (left panel) mean *R*^2^ was 0.65 with mean RMSE of 2.57. For the all data model (right panel) *R*^2^ of 0.45 and RMSE of 3.17 was observed.

## Discussion

We present a method to estimate surrogate measures of cognitive function, balance impairment, and falls risk by quantifying the FTSS test using sensors mounted on either the torso or the thigh, validated on a sample of in a sample of 168 community-dwelling older adults. A novel signal processing algorithm is used to extract features from either sensor, which was in turn used to examine statistical reliability, estimate clinical scores of balance and cognitive function (BBS and MMSE), and also to classify balance impairment, cognitive decline, and falls risk. We believe this study is the first to combine estimation of cognitive function, balance, and falls risk into a single test using wearable sensors. Furthermore, we believe it offers significant potential for use in remote, unsupervised screening of physical and cognitive function.

The causal relationship between cognitive function and functional test performance is not well understood. It is hypothesized that cognitive function could affect the performance in executing functional tests (such as the chair stand or FTSS tests), a variety of studies have found that low cognitive scores were associated with, and predicted poorer performance in a variety of functional tests (Carlson et al., 1999; Ble et al., 2005; Verghese et al., 2007; Annweiler et al., 2011; McGough et al., 2011). Previous research from our group (Greene and Kenny, 2012) found that change in gait observed using inertial sensors during the TUG test could be used to predict cognitive decline in community-dwelling older adults. To the best of our knowledge, this study reports the first method to estimate MMSE using a functional test or classify cognitive decline using inertial sensors and the FTSS test.

Similä et al. (2014) used a variety of BBS sub-tasks along with a gait task, instrumented with an accelerometer, to estimate BBS scores in 54 subjects, which included neurological patients, older adults, and healthy controls. Bacciu et al. (2017) used the Wii balance board combined with machine learning to estimate the BBS score on a sample of 21 older adults. To the best of our knowledge, the present study is the first to estimate BBS from the FTSS test. This study introduces a number of new features to quantify movement from a torso or thigh-mounted IMU during sit-to-stand tests, specifically intended to examine stand speed (mean of angular velocity and acceleration values at mid-stand, sit-end, and stand-start times), complexity (spectral entropy), and endurance (mean and standard deviation of angular velocity and acceleration values at mid-stand, sit-end and stand start times) during the FTSS test. These features along with other features reported elsewhere (Doheny et al., 2013) (such as sit-stand-sit time and RMS values) seek to quantify aspects of dynamic balance, strength, and endurance that cannot be measured using standard functional tests.

A number of previous studies (Doheny et al., 2013; Greene et al., 2014; van Lummel et al., 2016; Ejupi et al., 2017; Pham et al., 2018; Adamowicz et al., 2020) have investigated using the FTSS or other sit-to-stand tests to assess physical function using a variety of sensor placements including torso, thigh and lumbar, using a variety of approaches including adaptive peak detection and wavelets, in both free-living and clinical environments. For assessing falls risk, this study reports results in line with those reported previously but the results point to the potential for an assessment based on data from a single sensor placed in one of two different sensor locations. Previous results from our group (Greene et al., 2014) reported a classification accuracy of 72.63% (average across separate male/female models) in classifying falls risk using accelerometer data obtained from the thigh sensor during the FTSS and a Support Vector Machine (Vapnik, 1998) based on a sample of 124 older adults. The equivalent results from the present study were 65.33%, the reduced performance can perhaps be explained by heterogeneity arising from pooling of data from two distinct data sets along with the use of a simpler classifier model (logistic regression). Logistic regression was chosen as a simple, explainable method to demonstrate the effects apparent in the data, rather than a more complex classifier, which can be more difficult to interpret and more prone to overfitting. Furthermore, it is noteworthy that exploratory analysis did not detect any significant differences in FTSS parameters between fallers and non-fallers for the reported sample. With any studies using machine learning methods to develop predictive models based on wearable sensor data, there is a risk of over-optimization. To minimize the risk of bias, each of the statistical models reported here was validated using cross-validation, with features chosen within the cross-validation procedure to minimize bias in estimating performance on unseen data (Hastie et al., 2009). Furthermore, we have not reported confidence intervals for the reported cross-validated classification results due to the risk of statistical bias (Bengio and Grandvalet, 2004).

Exploratory analysis showed that those STS features based on postural sway, as well as those based on mean timing values showed good or excellent reliability. Those features based on coefficients of variation and jerk exhibited lower reliability. Moderate correlations were also observed between individual inertial sensor features and both MMSE and BBS, particularly for those features based on sit-to-stand timing and postural sway.

The ranges of MMSE and BBS values reported in this study suggested that participants were mainly higher performing, future work will seek to validate the reported results in more frail and cognitively impaired populations. Although widely employed in the clinical setting, both the BBS and MMSE scales have some limitations as measures of balance and cognitive function, respectively, owing to issues with ceiling effects in assessing balance (Donoghue and Stokes, 2009) and poor predictive validity in neuropsychological functioning (Faustman et al., 1990). Threshold values were chosen to dichotomize participants based on balance and cognitive impairment using threshold (cut-off) values recommended in the literature; However, a number of different cut-off values for both BBS and MMSE (Cullen et al., 2005; Muir et al., 2008; Donoghue and Stokes, 2009) have been reported depending on the population which could affect some of the results presented, depending on the threshold chosen. Retrospective self-reporting of falls was used; this can be problematic due to memory recall, particularly in an aging population. However, in this case, retrospective self-report was based on clinician-led questioning and not an independently completed survey instrument.

We report an algorithm that could support remote screening by estimating surrogate measures of balance impairment, cognitive decline and falls risk, based on the inertial sensor data and the FTSS test. Administering a single functional test has the benefit of reducing the time and complexity required for remote assessments; The FTSS is a relatively simple test to perform, with no physical space requirements beyond a standard chair; as such it may be suitable for unsupervised, remote assessment in the home. The algorithms presented here are intended to be applied to the sensors embedded in a smartphone (i.e., with the phone held against the torso during the test) and used as part of a more comprehensive platform for remote physical assessment, to aid with unsupervised, remote monitoring of balance, cognitive function and falls risk in the home.

## Data Availability

Restrictions apply to the availability of these data, which were used under license for the current study, and so are not publicly available. Data are however available from the authors upon reasonable request and with permission of University College Dublin and Kinesis Health Technologies Ltd.

## Supplementary Materials

To view supplementary material for this article, please visit http://doi.org/10.1017/wtc.2022.6.
